# Supplementary material for: Modulated Fluorescence in LB Films Based on DADQs—A Potential Sensing Surface?
Source: Molecules. 2022 Jun 17;27(12):3893. doi: 10.3390/molecules27123893 (PMC9229250; doi:10.3390/molecules27123893)
Supplement: Supplementary file 1 [file molecules-27-03893-s001.zip › molecules-1692034-supplementary.pdf]

molecules

Supplementary information - Sensing surfaces based on functionalised fluorescent dicynaoquinomethane Langmuir-Blodgett films.

Marek Szablewski [1], Richard L. Thompson [2] and Lars-Olof Pålsson [2,*]

[1] Department of Physics, Durham University DH1 3LE, UK; marek.szablewski@durham.ac.uk
[2] Department of Chemistry, Durham University, DH1 3LE, UK; richard.l.thompsonl@durham.ac.uk
* Correspondence: lars-olof.palsson@durham.ac.uk; Tel.: +44-(0)191-3342135

Synthetic procedures.

Three different amphiphilic DADQs (with single R1 functional groups structures) for LB film fabrication where used in this study; **1** (2-(4-(5-(hydroxymethyl)-1,3-oxazinan-2-ylidene)cyclohexa-2,5dien-1-ylidene)malononitrile), **2** (2-(4-(4-butyloxazolidin-2-ylidene)cyclohexa-2,5-dien-1ylidene)malononitrile) and **3** (2-(4-((hexylamino)(3-hydroxypyrrolidinol-1-yl)methylene)cyclohexa2,5-dien-1-ylidene)malononitrile). Synthetic procedures for 1,2 and 3 followed the methods outlined in (REF), for these type of adducts.

Compounds **1** and **2** were prepared by adding a slight excess of 2-(aminomethyl)-1,3-propanediol, or 2-amino-1-hexanol respectively to a warm solution of TCNQ in acetonitrile. The solution was stirred with heating for 2.5 hrs, and then left to cool slowly. The products were collected as yellow fibrous microcrystals by filtration by gravity. They were subsequently recrystallized from hot acetonitrile or methanol and dried in vacuo. Typically the yield was in the region of 50%.

Compound **1** 1H NMR, (d6DMSO), δ 3.4-3.6 ppm, broad singlet/multiplet and multiplet, heterocycle -CH2 (x2), δ 4.5-4.9 ppm, 3 multiplets, heterocycle -(CH)- and exocyclic -(CH)2- ,δ 4.4ppm, broad singlet (OH), δ 6.85 & 7.65 ppm, doublet of doublets, quinoidal ring –(CH) x4, δ 12.4 ppm (approx.) broad singlet (NH). λ_{max} (solvent) xxx nm, Microanalysis: Calculated for C14H13N3O2 : C, 65.87; N, 16.46; H, 5.13%. Found: C, 61.21; N, 16.47; H, 4.19 %

Compound **2**: -(620 mg) 48% yield. 1H NMR, (CD_2Cl_2) shows characteristic doublet of doublets centred around δ 7.2 ppm and N-H band, all aliphatic signals compressed. Mass spectrum: m/z, M+ 267. (100%, Molecular ion), ir: (KBr disc) υ(nitrile) 2179, 2132cm^{-1} , (characteristic doublet of C≡N stretch in such species),λ_{max} (acetone) 424nm, Mp/decomp temp approx. 230°C. Microanalysis: Calculated for C16H17N3O : C, 71.89; N, 15.72; H, 6.41%. Found: C, 71.75; N, 15.74; H, 6.37%.

Compound **3** was prepared in two stages, firstly a slight excess of 3-pyrrodinol was added to a solution of TCNQ in THF, when the solution turned purple, it was warmed to 50 C, and left for 20 hours, after which it was allowed to cool. The product was isolated as a

Commented [SM1]: Need to add lambda max from the students report.

Commented [SM2]: The micro analysis is not really v good, perhaps better not to include? Compare to how good compound 2 micro is. Really annoyed by this. Could try to do a MS that would fix it.

dark purple powder by filtration by gravity, and recrystallized from hot acetonitrile. The yield was typically in the region of 80%.

The purple powder prepared above was dissolved in warm THF and a large excess of hexylamine was added. The reaction was left at 30 C for 4 hours. After cooling, compound **3**, a light yellow powder, was collected by filtration, and recrystallized from ethyl acetate. The yield was typically in the region of 30%

Compound **3**

Optical spectroscopy.

Due to the low absorption cross-section it was very difficult to record absorption spectra of the LB films. Fluorescence spectra of the LB films could be recorded though, due to high sensitivity of the spectrometer used. The spectra are shown in figure S1. Fluorescence spectra were recorded using a Jobin Yvon Horiba Fluoromax 3. Microscope slides were mounted at an angle of ~ 45 degrees relative to normal of the incoming excitation light, facing the detection port. To minimise scattered light, the excitation slit width was set to 2.5 nm and the detection to 1.5 nm (band pass).

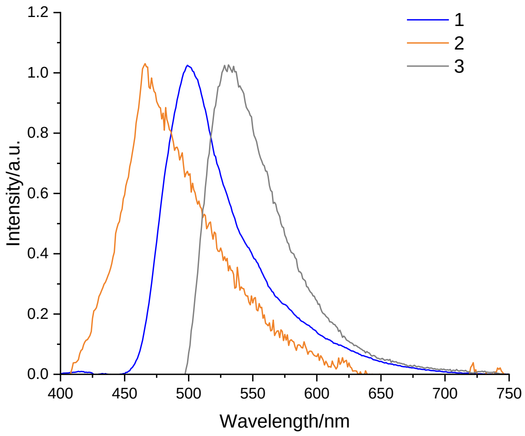

Figure S1. Normalised fluorescence spectra of pristine LB films of **1**, **2** and **3**. Excitation is at 385 nm. The number of deposited layers is 2.

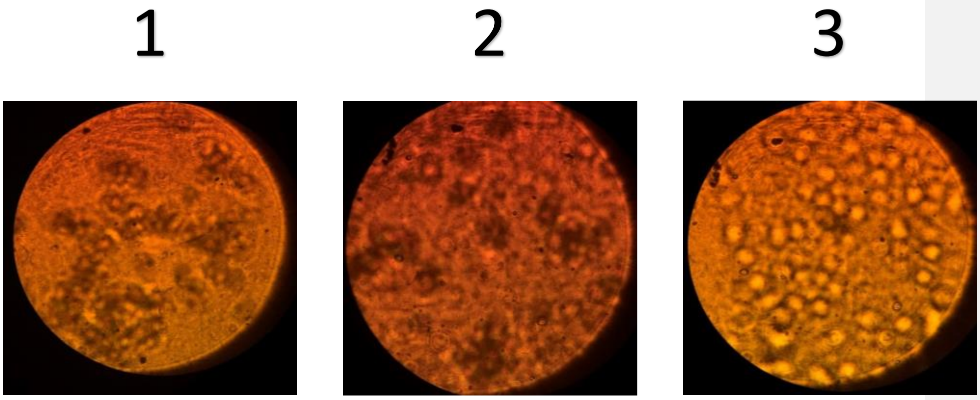

Figure S2. Normal camera images of the LB films of **1**, **2** and **3** (pristine/blank films) as viewed in the micrscope. The images are contrast images from a white light light source but filtered by a 595 nm longpass filter. The dimension of the images is a diamterer of 15 μm.

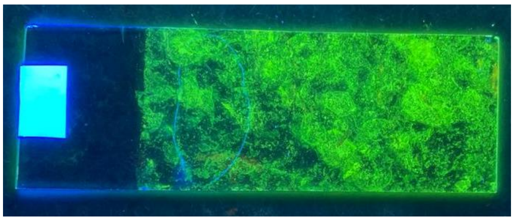

Figure S3. Fluorescence from a full sized microscope slide of the pristine LB film of **3** under UV excitation.

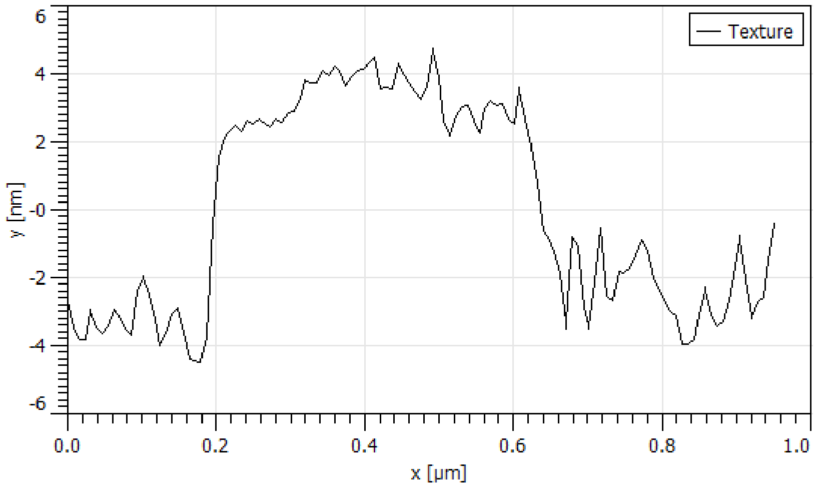

Figure S4. Cross-sectional AFM height profile of the texture for a droplet of HA deposition.

Table S1. The complete set data from the time-resolved fluorescence microscopy experiments for the data that is presented in figure 6 but without uncertainties. These were obtained through repeated measurements on a few different LB films surface points. Fitted fluorescence lifetimes and amplitudes form LB films of **1** in the two left columns. "top" and "den" (denominator) refer to the terms as defined in equation 2.

τ_i/ns	amp$_i$/a.u.	top	den	sum top	sum den	< τ > /ns	Chi sq
**1** blank							
2.5858	0.0594	0.39717	0.153597	0.551723	0.486008	1.135214	0.908
0.2147	0.6321	0.029137	0.135712				

0.6376	0.3085	0.125416	0.1967				
**1** NiO							
0.6699	0.2829	0.126956	0.189515	0.486074	0.465129	1.04503	0.908
0.2263	0.6696	0.034291	0.15153				
2.6178	0.0474	0.324826	0.124084				
**1** Bis A							
3.133	0.0104	0.102083	0.032583	0.121591	0.074315	1.636166	1.035
0.7239	0.03017	0.01581	0.02184				
0.1859	0.107	0.003698	0.019891				
**1** HA							
3.7202	1.001	13.83989	3.7202	76.81469	20.60284	3.728354	1.036
0.5699	0.0014	0.000455	0.000798				
3.7303	4.5256	62.97435	16.88185				
**1** HSA							
0.442	0.7594	0.148359	0.335655	1.018853	0.70192	1.451524	1.393
2.3892	0.3361	1.918552	0.80301				
2.3997	-0.182	-1.04806	-0.43675				

Table S2. The complete set data from the time-resolved fluorescence microscopy experiments for the data that is presented in figure 6 but without uncertainties. These were obtained through repeated measurements on a few different LB films surface points. Fitted fluorescence lifetimes and amplitudes form LB films of **2** in the two left columns. “top” and “den” (denominator) refer to the terms as defined in equation 2.

τ_i/ns	amp$_i$/a.u.	top	den	sum top	sum den	$<\tau>$ /ns	Chi sq
**2** blank							
0.458	0.174	0.036499	0.079692	17.40797	3.926276	4.433712	0.916
1.6235	0.4211	1.109915	0.683656				
5.1413	0.6152	16.26156	3.162928				

**2** NiO							
0.3149	0.2275	0.022559	0.07164	8.125253	2.237888	3.630768	0.872
1.3375	0.3728	0.666904	0.49862				
4.4589	0.374	7.435789	1.667629				
**2** Bis A							
0.081	0.176	0.001155	0.014256	0.695638	0.661641	1.051383	1.014
0.7442	0.7442	0.412163	0.553834				
3.0178	0.031	0.282321	0.093552				
**2** HA							
0.0324	5.17E-01	0.000543	0.016764	0.1748	0.076255	2.292314	0.994
0.6546	0.0298	0.012769	0.019507				
4.0388	9.90E-03	0.161488	0.039984				
**2** HSA							
0.0668	5.36E-02	0.000239	0.00358	0.706379	0.167842	4.2086	0.955
0.8081	0.0269	0.017566	0.021738				
4.8313	2.95E-02	0.688573	0.142523				

Table S3. The complete set data from the time-resolved fluorescence microscopy experiments for the data that is presented in figure 6 but without uncertainties. These were obtained through repeated measurements on a few different LB films surface points. Fitted fluorescence lifetimes and amplitudes form LB films of **3** in the two left columns. "top" and "den" (denominator) refer to the terms as defined in equation 2.

τ_i/ns	amp$_i$/a.u.	top	den	sum top	sum den	$< \tau >$ /ns	Chi sq
3 blank							
0.1574	0.4375	0.010839	0.068863	0.189762	0.200533	0.946287	0.904
0.486	0.1516	0.035807	0.073678				
2.4678	0.0235	0.143116	0.057993				

3 NiO							
0.0334	0.9687	0.001081	0.032355	0.850008	0.339244	2.505592	1.151
0.4523	0.1955	0.039994	0.088425				
3.7028	0.059	0.808933	0.218465				
3 Bis A							
0.0898	0.2838	0.002289	0.025485	0.104653	0.109065	0.959554	1.434
0.5784	0.0881	0.029474	0.050957				
2.2344	0.0146	0.072891	0.032622				
3 HA							
0.0478	3.11E-01	0.000709	0.014842	0.081233	0.064137	1.266564	0.956
0.5239	0.0515	0.014135	0.026981				
2.9752	7.50E-03	0.066389	0.022314				
3 HSA							
0.1331	0.8534	0.015119	0.113588	0.282611	0.310883	0.909058	0.947
0.4989	0.2089	0.051995	0.10422				
2.3153	0.0402	0.215497	0.093075				

molecules MDPI

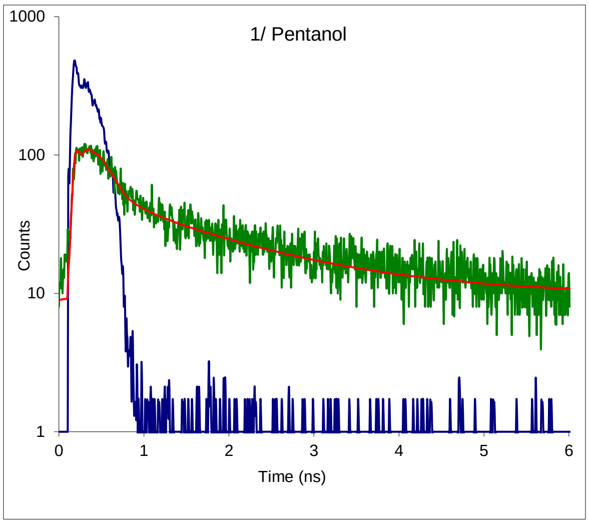

Amp1	Tau1 (ns)	Yield1 %
0.0020	0.2098	8.8280
Amp2	Tau2 (ns)	Yield 2 %
0.0021	1.5192	66.4782
Amp3	Tau3 (ns)	Yield 3 %
0.0448	0.0258	24.2739

Figure S5. The LB film of **1** after exposure of a droplet of pentanol. Green is raw data, orange is fitted function and blue is IRF used in the fitting procedure. Decay fitted to a sum of three exponentials and outcome from the fitting procedure shown in the table immediately below the figure of the decay. The kinetic component with a lifetime of ~26 ps (Tau 3 in this table) does not appear in the pristine LB film **1** or for LB film **1**s with any of the target analyte exposures. We therefore attribute this component to DADQ molecules in solution, detached from the surface due to pentanol exposure.

molecules

MDPI

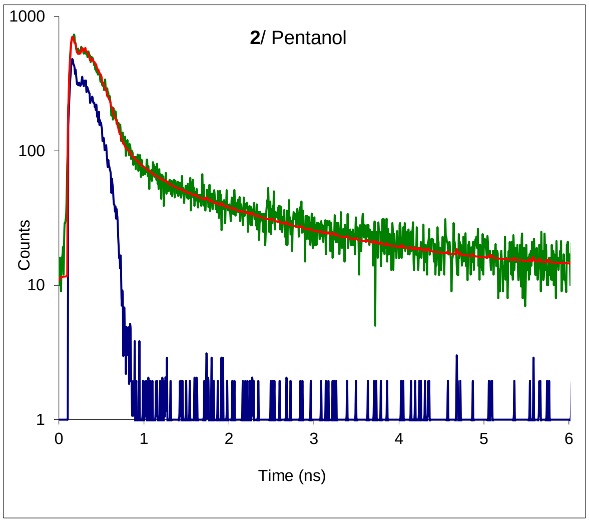

Amp1	Tau1 (ns)	Yield1 %
0.0089	0.2543	14.3633
Amp2	Tau2 (ns)	Yield 2 %
0.0036	1.4974	34.4070
Amp3	Tau3 (ns)	Yield 3 %
1.1672	0.0069	51.2297

Figure S6. The LB film of **2** after exposure of a droplet of pentanol. Green is raw data, orange is fitted function and blue is IRF used in the fitting procedure. Decay fitted to a sum of three exponentials and outcome from the fitting procedure shown in the table immediately below the figure of the decay. The kinetic component with a lifetime of ~70 ps has the highest yield of all three decay phases as seen in the table above, and higher than what was observed for LB films of 2 with HSA exposure. We therefore attribute this component to DADQ molecules in solution, detached from the surface due to pentanol exposure.

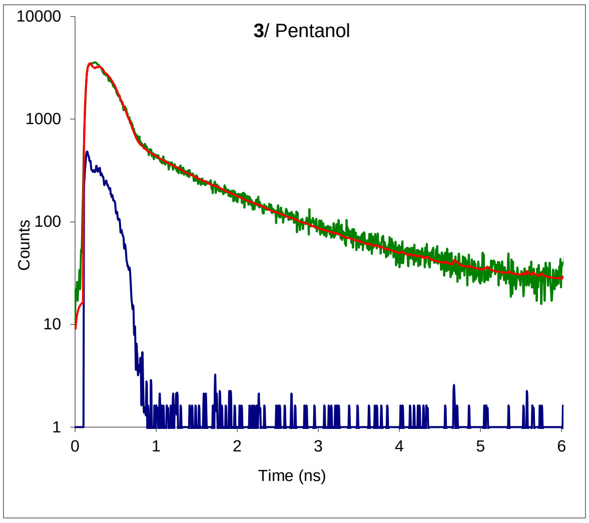

Amp1	Tau1 (ns)	Yield1 %
0.030467022	1.160115106	37.7571
Amp2	Tau2 (ns)	Yield 2 %
0.015121123	0.401018357	6.4776
Amp3	Tau3 (ns)	Yield 3 %
1.643847351	0.031756668	55.7653

Figure S7. The LB film of **3** after exposure of a droplet of pentanol. Green is raw data, orange is fitted function and blue is IRF used in the fitting procedure. Decay fitted to a sum of three exponentials and outcome from the fitting procedure shown in the table immediately below the figure of the decay.
